# The ICECAP Study Did Not Find Evidence for Better Outcomes With Prolonged Cooling After Out‐Of‐Hospital Cardiac Arrest

**DOI:** 10.1111/aas.70337

**Published:** 2026-09-15

**Authors:** Anders Aneman, Niklas Nielsen, Markus B. Skrifvars

**Affiliations:** ^1^ Intensive Care Unit Liverpool Hospital, South Western Sydney Local Health District Sydney Australia; ^2^ Department of Clinical Sciences Lund University Lund Sweden; ^3^ Anaesthesia and Intensive Care Helsingborg Hospital Lund Sweden; ^4^ Clinical Studies Sweden ‐ Forum South Lund Sweden; ^5^ Department of Anaesthesiology and Intensive Care Medicine Helsinki University Hospital and University of Helsinki Helsinki Finland

Hypoxic–ischaemic brain injury is a devastating condition and the leading cause of death in patients resuscitated after cardiac arrest [1]. Although supportive post‐cardiac‐arrest care and neurological prognostication have evolved, a therapeutic approach to limit established hypoxic–ischaemic brain injury remains elusive, with management currently focused on preventing secondary injury and treating complications.

Following initial encouraging results from induced hypothermia to 33°C, more recent and larger studies have failed to demonstrate an improvement in either survival or functional neurological outcome with hypothermic temperature management. Considerable practice variation exists in how temperature management is applied following cardiac arrest. Current guidelines recommend maintaining temperature control between 32°C and 37.5°C for at least 36 h in comatose survivors [2] or emphasise active fever prevention, generally for 36–72 h, rather than a prescribed hypothermic target [3]. Most trials that informed these guidelines investigated hypothermia for 24 h followed by slow rewarming. However, animal experimental evidence has suggested that prolonged hypothermia for 48 h may provide greater neuroprotection [4]. Cerebral oedema following hypoxic–ischaemic brain injury typically develops during the first days after cardiac arrest. Rewarming while cerebral oedema is evolving might therefore theoretically be disadvantageous, considering the effects of hypothermia on cerebral metabolism and intracranial pressure. The TTH48 trial [5] compared hypothermia at 33°C for 24 versus 48 h and did not demonstrate any benefit from prolonged hypothermia, while underpowered to establish the significance of the 5% higher rate of good outcome in the 48‐h group.

The results of the Influence of Cooling Duration on Efficacy in Cardiac Arrest Patients (ICECAP) trial were recently published [6]. The ICECAP study delivers one clear and immediately actionable message. In comatose survivors of out‐of‐hospital cardiac arrest who have already been cooled rapidly to 33°C, extending hypothermia beyond the shortest duration of 6 h investigated, does not improve neurological recovery. Clinicians using device‐controlled hypothermia for 48 h on the strength of preclinical data and TTH48 would find no support with this evidence added. The ICECAP trial rewards careful reading, with design features making it a methodologically innovative and important contribution to cardiac arrest research, although these arguably also constrain what may be concluded.

The ICECAP study was an investigator‐initiated, Bayesian, response‐adaptive duration‐finding trial conducted at 71 US academic and community hospitals between June 2020 and June 2025. Comatose adults after out‐of‐hospital cardiac arrest were eligible if a definitive closed‐loop temperature‐control device had been started, core temperature was below 34°C within 4 h of arrest, and randomisation could occur within 6 h of device activation. Patients with severe haemodynamic instability and those with major limitations of care were excluded. The primary outcome was a weighted 90‐day modified Rankin Scale (mRS) score from 0 to 10, higher score indicating better outcome, designed to enhance nuances around favourable outcomes and in which standard mRS 4, 5 and 6 all scored zero (Figure 1A). The first 200 participants were allocated 1:1:1 to 12, 24 or 48 h of hypothermia, after which an adaptive algorithm allocated preferentially to durations likely to be optimal, separately within patients with shockable and non‐shockable initial rhythm. The allocation was based on a pre‐defined statistical response model that was able to fit many shapes of the duration‐response curve (increasing, decreasing, flat or U‐shaped, Figure 1B) and all conclusions about the treatment arms were based on this model. A 6‐h duration arm was opened since the emerging duration‐response curve was flat, that is, the lack of benefit from longer duration prompted the inclusion of a shorter duration arm to minimise exposure to a non‐beneficial intervention not without its risks. Similarly, 60‐ and 72‐h duration arms could have been opened had the duration‐response curve showed signs of increasing benefit, but this was never the case. The primary aim of the study was to determine the shortest duration of hypothermia targeting 33°C that provided the maximum treatment effect. The study found no signal of benefit from longer durations of cooling in the weighted mRS (Figure 1C) mortality, dichotomised mRS, neuropsychological testing, adverse events in any initial rhythm cohort or in any prespecified subgroup (including age, comorbidities, likely cause of arrest, cooling device, pre‐hospital adrenaline dose, ST‐elevation, ischaemic findings on initial CT). It was stopped after the inclusion of 1158 patients based on a prespecified interim stopping criterion (posterior probability 0.5 threshold, Figure 1D). With a flat fitted duration‐response curve, the posterior probability accumulates at the shortest duration permitted by the study design. The posterior probability near 0.5 is hence not evidence that 6 h is optimal, and approximately 0.30 of the posterior was apportioned 12 h. The secure conclusion is that longer duration does not convey benefit, with 0.013 of the posterior apportioned 48 h. Importantly, the study included patients with profound forms of cardiac arrest, with a majority presenting with a non‐shockable initial rhythm. Thus, the study provides important new information on a group of patients who have been less well represented in previous large trials of temperature management.

**FIGURE 1:**
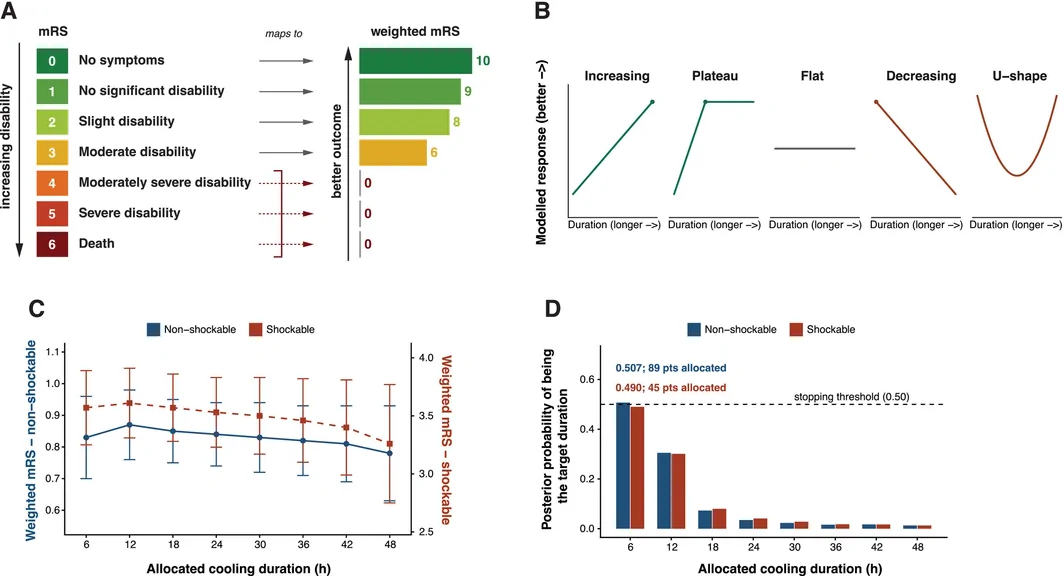
Presentation of the mRS scoring used (A), the potential duration‐response curves (B), the weighted mRS outomes (C) and the distribution of posterior probabilities for the target duration (D).

The study prompts several interesting questions. The time from cardiac arrest to achieving the target temperature appears short and may seem shorter than in previous trials. Restricting inclusion to patients that reached a target below 34°C within 4 h of cardiac arrest was based on the rationale that if hypothermia is to provide neuroprotection, it must be achieved as early as possible. Notwithstanding, patients in the 6‐h group spent most of the time moving down to and up from the target temperature of 33°C. Perhaps the only way to achieve stable hypothermia very early is to initiate cooling during cardiac arrest, that is currently being investigated in the PRINCESS2 trial [7]. It is noteworthy that out of 13,112 patients assessed for eligibility, 9367 (71%) patients were excluded since not reaching the target temperature within 4 h, often because of the lack of an available cooling device. Most of the patients included in ICECAP had an initial non‐shockable rhythm, representing a group with much worse outcome compared to patients with a shockable rhythm [8]. The findings therefore need to be interpreted in the context of this highly selected population. Patients who are easier to cool may also be those with a more severe brain injury and more deranged thermoregulation, a group with overall poorer outcomes and lesser chance of benefit from any therapeutic intervention including induced hypothermia Similar rates of survival to discharge from hospital in enrolled (28%) and in screened but not enrolled (30.6%) patients argue to a degree against this potential selection bias.

The study was planned before the results of the TTM2 trial became available, but in retrospect one cannot help regretting that a normothermic control group was not included. Such a group would have been valuable in determining whether even a short period of hypothermia improves outcome compared to normothermia. The efficacy of hypothermia compared to no cooling was in ICECAP designed to be inferred from a positive duration‐response curve, thus avoiding a normothermia arm that could have been difficult to establish equipoise for at sites where cooling was already a clinical routine. The flat duration‐response curve defeats any such inference since it is equally consistent with ‘short hypothermia is enough with no added benefit from longer duration’ as with ‘hypothermia provides no benefit at any of the durations studied’. It is important to note that actively maintaining normothermia until mechanical ventilation was ceased or up to 120 h was applied in all patients, and hence treatment based on the TTM2‐evidence remains unchanged [9]. The role of temperature‐control devices specifically for the prevention and treatment of fever following OHCA is investigated in the ongoing STEPCARE trial [10]. Indeed, many questions about fever after cardiac arrest remains, including how common it is and is it amenable to treatment [11].

Finally, withdrawal of life‐sustaining therapy (WLST) preceded death in 59% and 38% of patients with non‐shockable and shockable initial rhythm, respectively. This occurred within 96 h in 16% of patients with non‐shockable rhythm and in 11% of patients with shockable rhythm despite the study protocol stating that neurological prognostication should be deferred beyond this timepoint. As treating teams and families were unblinded to the allocated duration given the nature of the intervention, the proportion of early deaths following withdrawal decisions is not a minor limitation. Recent studies have emphasised the effect of WLST on outcome in brain injury patients [12]. This highlights the importance of the robust blinded procedures for neurological prognostication applied in TTM and TTM2 and the ongoing STEPCARE trials.

Placed alongside TTM, TTM2 and the individual‐patient meta‐analysis of non‐shockable participants from HYPERION and TTM2 [13], the balance of evidence on whether to induce hypothermia at all has not moved. It will be interesting to see how these findings from the ICECAP study influence future international recommendations.

## Author Contributions

All authors contributed equally to the conception and writing of this editorial.

## Funding

The authors have nothing to report.

## Conflicts of Interest

The authors declare no conflicts of interest.

## Data Availability

Data sharing not applicable to this article as no datasets were generated or analysed during the current study.
